# Gender differences in the use of cardiovascular interventions in HIV‐positive persons; the D:A:D Study

**DOI:** 10.1002/jia2.25083

**Published:** 2018-03-06

**Authors:** Camilla I Hatleberg, Lene Ryom, Wafaa El‐Sadr, Amanda Mocroft, Peter Reiss, Stephane De Wit, Francois Dabis, Christian Pradier, Antonella d'Arminio Monforte, Helen Kovari, Matthew Law, Jens D Lundgren, Caroline A Sabin, the Data Collection of Adverse Events of Anti‐HIV drugs (D:A:D) Study group, G Calvo, F Bonnet, O Kirk, L Morfeldt, R Weber, A Lind‐Thomsen, R Salbøl Brandt, M Hillebreght, S Zaheri, FWNM Wit, A Scherrer, F Schöni‐Affolter, M Rickenbach, A Tavelli, I Fanti, O Leleux, J Mourali, F Le Marec, E Boerg, E Thulin, A Sundström, G Bartsch, G Thompsen, C Necsoi, M Delforge, E Fontas, C Caissotti, S Mateu, F Torres, K Petoumenos, A Blance, R Huang, R Puhr, K Laut, D Kristensen, AN Phillips, DA Kamara, CJ Smith, RS Brandt, D Raben, C Matthews, A Bojesen, AL Grevsen, B Powderly, N Shortman, C Moecklinghoff, G Reilly, X Franquet, C Smit, M Ross, CA Fux, P Morlat, N Friis‐Møller, J Kowalska, J Bohlius, M Bower, G Fätkenheuer, A Grulich, A Sjøl, P Meidahl, JS Iversen, M Hillebregt, JM Prins, TW Kuijpers, HJ Scherpbier, JTM van der Meer, MH Godfried, T van der Poll, FJB Nellen, SE Geerlings, M van Vugt, D Pajkrt, JC Bos, WJ Wiersinga, M van der Valk, A Goorhuis, JW Hovius, J van Eden, A Henderiks, AMH van Hes, M Mutschelknauss, HE Nobel, FJJ Pijnappel, S Jurriaans, NKT Back, HL Zaaijer, B Berkhout, MTE Cornelissen, CJ Schinkel, XV Thomas, A De Ruyter Ziekenhuis, M van den Berge, A Stegeman, S Baas, L Hage de Looff, C Ziekenhuis, MJH Pronk, HSM Ammerlaan, E de Munnik, J Tjhie, MCA Wegdam, B Deiman, V Scharnhorst, AM Weijsenfeld, ME van der Ende, ECM van Gorp, CAM Schurink, JL Nouwen, A Verbon, BJA Rijnders, HI Bax, M van der Feltz, N van der Bassant, JEA van Beek, M Vriesde, LM van Zonneveld, A de Oude‐Lubbers, HJ van den Berg‐Cameron, FB Bruinsma‐Broekman, J de Groot, M de Zeeuw‐de Man, CAB Boucher, MPG Koopmans, JJA van Kampen, SD Pas, GJA Driessen, AMC van Rossum, LC van der Knaap, E Flevoziekenhuis, J Branger, A Rijkeboer‐Mes, EF Schippers, JM van IJperen, J Geilings, G van der Hut, PFH Franck, A van Eeden, W Brokking, M Groot, LJM Elsenburg, M Damen, IS Isala, PHP Groeneveld, JW Bouwhuis, JF den Berg, AGW van Hulzen, GL van der Bliek, PCJ Bor, P Bloembergen, MJHM Wolfhagen, GJHM Ruijs, FP Kroon, MGJ de Boer, MP Bauer, H Jolink, AM Vollaard, W Dorama, N van Holten, ECJ Claas, E Wessels, JG den Hollander, K Pogany, A Roukens, M Kastelijns, JV Smit, E Smit, D Struik‐Kalkman, C Tearno, M Bezemer, T van Niekerk, O Pontesilli, SH Lowe, AML Oude Lashof, D Posthouwer, RP Ackens, J Schippers, R Vergoossen, B Weijenberg‐Maes, IHM van Loo, TRA Havenith, EMS Leyten, LBS Gelinck, A van Hartingsveld, C Meerkerk, GS Wildenbeest, JAEM Mutsaers, CL Jansen, JW Mulder, SME Vrouenraets, FN Lauw, MC van Broekhuizen, H Paap, DJ Vlasblom, PHM Smits, S Weijer, R El Moussaoui, AS Bosma, MGA van Vonderen, DPF van Houte, LM Kampschreur, K Dijkstra, S Faber, J Weel, GJ Kootstra, CE Delsing, M van der Burg‐van de Plas, H Heins, E Lucas, W Kortmann, G van Twillert¤, JWT Cohen Stuart, BMW Diederen, D Pronk, FA van Truijen‐Oud, WA van der Reijden, R Jansen, K Brinkman¤, GEL den Berk, WL Blok, PHJ Frissen, KD Lettinga, WEM Schouten, J Veenstra, CJ Brouwer, GF Geerders, K Hoeksema, MJ Kleene, IB van der Meché, M Spelbrink, H Sulman, AJM Toonen, S Wijnands, D Kwa, E Witte, PP Koopmans, M Keuter, AJAM van der Ven, HJM ter Hofstede, ASM Dofferhoff, R van Crevel, M Albers, MEW Bosch, KJT Grintjes‐Huisman, BJ Zomer, FF Stelma, J Rahamat‐Langendoen, D Burger, C Richter, EH Gisolf, RJ Hassing, G ter Beest, PHM van Bentum, N Langebeek, R Tiemessen, CMA Swanink, SFL van Lelyveld, R Soetekouw, N Hulshoff, LMM van der Prijt, J van der Swaluw, N Bermon, BL Herpers, D Veenendaal, DWM Verhagen, M van Wijk, AE Brouwer, M Kuipers, RMWJ Santegoets, B van der Ven, JH Marcelis, AGM Buiting, PJ Kabel, WFW Bierman, H Scholvinck, KR Wilting, Y Stienstra, PA van der Meulen, DA de Weerd, J Ludwig‐Roukema, HGM Niesters, A Riezebos‐Brilman, CC van Leer‐Buter, M Knoester, AIM Hoepelman, T Mudrikova, PM Ellerbroek, JJ Oosterheert, JE Arends, RE Barth, MWM Wassenberg, EM Schadd, DHM van Elst‐Laurijssen, EEB van Oers‐Hazelzet, S Vervoort, M van Berkel, R Schuurman, F Verduyn‐Lunel, AMJ Wensing, EJG Peters, MA van Agtmael, M Bomers, J de Vocht, M Heitmuller, LM Laan, AM Pettersson, CW Ang, SPM Geelen, TFW Wolfs, LJ Bont, DO Bezemer, AI van Sighem, TS Boender, A de Jong, D Bergsma, P Hoekstra, A de Lang, S Grivell, A Jansen, MJ Rademaker, M Raethke, R Meijering, S Schnörr, L de Groot, M van den Akker, Y Bakker, E Claessen, A El Berkaoui, J Koops, E Kruijne, C Lodewijk, L Munjishvili, B Peeck, C Ree, R Regtop, Y Ruijs, T Rutkens, L van de Sande, M Schoorl, A Timmerman, E Tuijn, L Veenenberg, S van der Vliet, A Wisse, T Woudstra, B Tuk, M Dupon, V Gaborieau, D Lacoste, D Malvy, P Mercié, D Neau, JL Pellegrin, S Tchamgoué, E Lazaro, C Cazanave, M Vandenhende, MO Vareil, Y Gérard, P Blanco, S Bouchet, D Breilh, H Fleury, I Pellegrin, G Chêne, R Thiébaut, L Wittkop, S Lawson‐Ayayi, A Gimbert, S Desjardin, L Lacaze‐Buzy, V Petrov‐Sanchez, K André, N Bernard, O Caubet, L Caunegre, I Chossat, C Courtault, FA Dauchy, D Dondia, P Duffau, H Dutronc, S Farbos, I Faure, H Ferrand, Y Gerard, C Greib, M Hessamfar, Y Imbert, P Lataste, J Marie, M Mechain, E Monlun, A Ochoa, T Pistone, I Raymond, MC Receveur, P Rispal, L Sorin, C Valette, MA Vandenhende, JF Viallard, H Wille, G Wirth, ME Lafon, P Trimoulet, P Bellecave, C Tumiotto, F Haramburu, G Miremeont‐Salamé, MJ Blaizeau, M Decoin, C Hannapier, E Lenaud et A Pougetoux, S Delveaux, C D'Ivernois, F Diarra, B Uwamaliya‐Nziyumvira, G Palmer, V Conte, V Sapparrart, R Moore, S Edwards, J Hoy, K Watson, N Roth, H Lau, M Bloch, D Baker, A Carr, D Cooper, M O'Sullivan, D Nolan, G Guelfi, P Domingo, MA Sambeat, J Gatell, E Del Cacho, J Cadafalch, M Fuster, C Codina, G Sirera, A Vaqué, N Clumeck, AF Gennotte, M Gerard, K Kabeya, D Konopnicki, A Libois, C Martin, MC Payen, P Semaille, Y Van Laethem, J Neaton, E Krum, G Thompson, R Luskin‐Hawk, E Telzak, DI Abrams, D Cohn, N Markowitz, R Arduino, D Mushatt, G Friedland, G Perez, E Tedaldi, E Fisher, F Gordin, LR Crane, J Sampson, J Baxter, B Gazzard, A Horban, I Karpov, M Losso, C Pedersen, M Ristola, A Phillips, J Rockstroh, L Peters, AH Fischer, K Grønborg Laut, JF Larsen, D Podlekareva, A Cozzi‐Lepri, L Shepherd, A Schultze, S Amele, M Kundro, B Schmied, A Vassilenko, VM Mitsura, D Paduto, E Florence, L Vandekerckhove, V Hadziosmanovic, J Begovac, L Machala, D Jilich, G Kronborg, T Benfield, J Gerstoft, T Katzenstein, NF Møller, L Ostergaard, L Wiese, LN Nielsen, K Zilmer, I Aho, J‐P Viard, P‐M Girard, C Duvivier, O Degen, HJ Stellbrink, C Stefan, J Bogner, N Chkhartishvili, P Gargalianos, J Szlávik, M Gottfredsson, F Mulcahy, I Yust, D Turner, M Burke, E Shahar, G Hassoun, H Elinav, M Haouzi, D Elbirt, ZM Sthoeger, R Esposito, I Mazeu, C Mussini, F Mazzotta, A Gabbuti, V Vullo, M Lichtner, M Zaccarelli, A Antinori, R Acinapura, M Plazzi, A Lazzarin, A Castagna, N Gianotti, M Galli, A Ridolfo, B Rozentale, V Uzdaviniene, T Staub, V Ormaasen, A Maeland, J Bruun, B Knysz, J Gasiorowski, M Inglot, E Bakowska, R Flisiak, A Grzeszczuk, M Parczewski, K Maciejewska, B Aksak‐Was, M Beniowski, E Mularska, T Smiatacz, M Gensing, E Jablonowska, E Malolepsza, K Wojcik, I Mozer‐Lisewska, L Caldeira, R Radoi, A Panteleev, A Yakovlev, T Trofimora, I Khromova, E Kuzovatova, E Borodulina, E Vdoushkina, D Jevtovic, J Tomazic, JM Gatell, JM Miró, S Moreno, JM Rodriguez, B Clotet, A Jou, R Paredes, C Tural, J Puig, I Bravo, M Gutierrez, G Mateo, JM Laporte, K Falconer, A Thalme, A Sonnerborg, A Blaxhult, L Flamholc, M Cavassini, A Calmy, H Furrer, P Schmid, A Kuznetsova, G Kyselyova, M Sluzhynska, AM Johnson, E Simons, MA Johnson, C Orkin, J Weber, G Scullard, A Clarke, C Leen, G Thulin, B Åkerlund, K Koppel, A Karlsson, C Håkangård, F Castelli, R Cauda, G Di Perri, R Iardino, G Ippolito, GC Marchetti, CF Perno, F von Schloesser, P Viale, F Ceccherini‐Silberstein, E Girardi, S Lo Caputo, M Puoti, M Andreoni, A Ammassari, C Balotta, A Bandera, P Bonfanti, S Bonora, M Borderi, A Calcagno, L Calza, MR Capobianchi, A Cingolani, P Cinque, A De Luca, A Di Biagio, A Gori, G Guaraldi, G Lapadula, G Madeddu, F Maggiolo, G Marchetti, S Marcotullio, L Monno, S Nozza, E Quiros Roldan, R Rossotti, S Rusconi, MM Santoro, A Saracino, L Galli, P Lorenzini, A Rodano, M Shanyinde, F Carletti, S Carrara, A Di Caro, S Graziano, F Petrone, G Prota, S Quartu, S Truffa, A Giacometti, A Costantini, V Barocci, G Angarano, C Santoro, C Suardi, V Donati, G Verucchi, C Minardi, T Quirino, C Abeli, PE Manconi, P Piano, B Cacopardo, B Celesia, J Vecchiet, K Falasca, A Pan, S Lorenzotti, L Sighinolfi, D Segala, F Vichi, G Cassola, C Viscoli, A Alessandrini, N Bobbio, G Mazzarello, C Mastroianni, V Belvisi, I Caramma, A Chiodera, P Milini, G Rizzardini, MC Moioli, R Piolini, AL Ridolfo, S Salpietro, C Tincati, C Puzzolante, N Abrescia, A Chirianni, G Borgia, R Orlando, G Bonadies, F Di Martino, I Gentile, L Maddaloni, AM Cattelan, S Marinello, A Cascio, C Colomba, F Baldelli, E Schiaroli, G Parruti, F Sozio, G Magnani, MA Ursitti, A Cristaudo, G Baldin, M Capozzi, S Cicalini, L Fontanelli Sulekova, G Iaiani, A Latini, I Mastrorosa, MM Plazzi, S Savinelli, A Vergori, M Cecchetto, F Viviani, P Bagella, B Rossetti, A Franco, R Fontana Del Vecchio, D Francisci, C Di Giuli, P Caramello, GC Orofino, M Sciandra, M Bassetti, A Londero, G Pellizzer, V Manfrin, G Starnini, A Ialungo, K Dollet, P Dellamonica, E Bernard, J Courjon, E Cua, F De Salvador‐Guillouet, J Durant, C Etienne, S Ferrando, V Mondain‐Miton, A Naqvi, I Perbost, S Pillet, B Prouvost‐Keller, P Pugliese, V Rio, K Risso, PM Roger, V Aubert, M Battegay, E Bernasconi, J Böni, DL Braun, Hc Bucher, A Ciuffi, G Dollenmaier, M Egger, L Elzi, J Fehr, J Fellay, HF Günthard, D Haerry, B Hasse, HH Hirsch, M Hoffmann, I Hösli, C Kahlert, L Kaiser, O Keiser, T Klimkait, RD Kouyos, B Ledergerber, G Martinetti, B Martinez de Tejada, C Marzolini, KJ Metzner, N Müller, D Nicca, G Pantaleo, P Paioni, A Rauch, C Rudin, AU Scherrer, R Speck, M Stöckle, P Tarr, A Trkola, P Vernazza, G Wandeler, S Yerly

**Affiliations:** ^1^ Department of Infectious Diseases Section 2100 CHIP University of Copenhagen Finsencentret Rigshospitalet Copenhagen Denmark; ^2^ ICAP‐Columbia University and Harlem Hospital New York NY USA; ^3^ Institute for Global Health UCL London United Kingdom; ^4^ Academic Medical Center Department of Global Health and Division of Infectious Diseases University of Amsterdam HIV Monitoring Foundation Amsterdam The Netherlands; ^5^ Division of Infectious Diseases Saint Pierre University Hospital Université Libre de Bruxelles Brussels Belgium; ^6^ CHU de Bordeaux and INSERM U897 Université de Bordeaux Talence France; ^7^ Department of Public Health Nice University Hospital Nice France; ^8^ Dipartimento di Scienze della Salute Clinica di Malattie Infettive e Tropicali Azienda Ospedaliera‐Polo Universitario San Paolo Milan Italy; ^9^ Division of infectious diseases and hospital epidemiology University hospital Zurich University of Zurich Zurich Switzerland; ^10^ Kirby Institute UNSW Sydney Sydney Australia

**Keywords:** Cardiovascular disease, gender, cardiovascular disease interventions, cohort studies, HIV, women, myocardial infarction, stroke

## Abstract

**Introduction:**

There is paucity of data related to potential gender differences in the use of interventions to prevent and treat cardiovascular disease (CVD) among HIV‐positive individuals. We investigated whether such differences exist in the observational D:A:D cohort study.

**Methods:**

Participants were followed from study enrolment until the earliest of death, six months after last visit or February 1, 2015. Initiation of CVD interventions [lipid‐lowering drugs (LLDs), angiotensin‐converting enzyme inhibitors (ACEIs), anti‐hypertensives, invasive cardiovascular procedures (ICPs) were investigated and Poisson regression models calculated whether rates were lower among women than men, adjusting for potential confounders.

**Results:**

Women (n = 12,955) were generally at lower CVD risk than men (n = 36,094). Overall, initiation rates of CVD interventions were lower in women than men; LLDs: incidence rate 1.28 [1.21, 1.35] vs. 2.40 [2.34, 2.46]; ACEIs: 0.88 [0.82, 0.93] vs. 1.43 [1.39, 1.48]; anti‐hypertensives: 1.40 [1.33, 1.47] vs. 1.72 [1.68, 1.77] and ICPs: 0.08 [0.06, 0.10] vs. 0.30 [0.28, 0.32], and this was also true for most CVD interventions when exclusively considering periods of follow‐up for which individuals were at high CVD risk. In fully adjusted models, women were less likely to receive CVD interventions than men (LLDs: relative rate 0.83 [0.78, 0.88]; ACEIs: 0.93 [0.86, 1.01]; ICPs: 0.54 [0.43, 0.68]), except for the receipt of anti‐hypertensives (1.17 [1.10, 1.25]).

**Conclusion:**

The use of most CVD interventions was lower among women than men. Interventions are needed to ensure that all HIV‐positive persons, particularly women, are appropriately monitored for CVD and, if required, receive appropriate CVD interventions.

## 1 Introduction

HIV‐positive individuals are known to be at increased risk of cardiovascular disease (CVD) compared to the general population 1, 2, partly due to an increased prevalence of some CVD risk factors, exposure to some antiretroviral drugs and chronic immune activation 3, 4, 5, 6, 7. Previous findings also indicate that HIV‐infection has a slightly greater impact on the risk of myocardial infarction (MI) and stroke in women compared to men 2, 3, 4, 8, 9.

In the general population, it is recognized that the risk of CVD, including MI and stroke, increases with age, and that women are less likely to develop CVD at any given age than men 10, 11, 12, 13, 14. However, this gender gap in cardiovascular morbidity diminishes with increasing age, as the protective effect of oestrogen wanes post‐menopause resulting in an increase in CVD morbidity in women 10, 11, 12, 13, 14, 15, 16. Although there have been substantial reductions in the incidence of MI and improvements in survival after MI and stroke over the last two decades 16, 17, 18, these improvements have lagged behind in women compared to men 16, 19, 20, 21. In particular, women have been shown to have higher in‐hospital mortality after MI at a younger age than men 22, 23, 24, 25, 26, 27, as well as higher rates of complications and mortality after invasive coronary interventions 28, 29. Evidence also indicates that women experience more severe stroke events, longer hospital stays and higher mortality rates following a stroke compared to men 16.

The reasons for these poorer outcomes remain unclear, but it is likely that multiple factors play a role, both before and after an event. Importantly, there is increasing evidence of delayed or less intensive use of medical and invasive procedures for diagnostic evaluation and treatment of MI and stroke among women compared to men 11, 12, 16, 20, 30, 31, 32, 33, 34. A previous study in the general population also demonstrated an inverse association between in‐hospital mortality after MI and the number of CVD risk factors that were present in an individual 35. This suggests that some individuals at apparently low CVD risk may have other, as yet unidentified, underlying risk factors or pathophysiological features that result in an MI of greater severity, a poorer prognosis and/or less optimal medical management. This hypothesis may pertain particularly to women, as they are generally perceived to be at lower risk of CVD, particularly if pre‐menopausal.

As in the general population, guidelines for the prevention of CVD among HIV‐positive individuals generally focus on groups at high CVD risk 36, 37, 38. Since women are generally considered to be at low CVD risk, it may be that the poorer outcomes in low risk individuals observed in the general population 35 may take on greater relevance among HIV‐positive women who are generally at higher overall risk of MI and stroke due to their HIV status 2, 3, 4, 7, 8, 9. An understanding of the use of interventions to prevent and treat CVD in HIV‐positive women compared to men is therefore required. The aim of this study was to investigate potential gender differences in the use of CVD‐related interventions in the large, prospective Data on Adverse effects of antiretroviral Drugs (D:A:D) study.

## 2 Methods

The D:A:D study is a large, prospective cohort study which follows >49,000 HIV‐positive persons from 11 collaborating cohorts in Europe, USA and Australia, contributing to >430,000 person years of follow‐up (PYRS). The details of the study have been described previously 6. The data are obtained prospectively with information collected on demographic factors, AIDS events, CD4 counts, HIV RNA viral loads, other laboratory test results (e.g. total cholesterol (TC), triglycerides (TG)), antiretroviral therapy‐regimen and treatment history, CVD risk factors and treatments. Data on clinical endpoints including non‐fatal/fatal MIs, strokes, deaths (including sudden cardiac death), and invasive cardiovascular procedures (ICPs; including coronary artery bypass grafts (CABGs), angioplasties and carotid endarterectomies) are reported to the D:A:D coordinating centre via designated case report forms and centrally validated according to standardized algorithms (https://www.chip.dk/Portals/0/files/Study%20documents/DAD_MOOP_revised2013.pdf). MIs are classified with a Dundee score using criteria from the WHO MONICA Study 39 and stroke events are validated on the basis of the presence of focal neurological signs with duration > 24 hours with no evidence of any non‐vascular cause. Causes of death are classified using the Coding of Causes of Death (CoDe) methodology, developed for the classification of causes of death in HIV‐positive persons (www.chip.dk/code) 40. This analysis was conducted in accordance with the Declaration of Helsinki, with approval by national ethics committees and informed consent where required by national regulations.

### 2.1 Statistical methods

Men and women were followed from baseline (date of entry into the D:A:D study which occurred on or after February 1, 1999) until the earliest of death, six months after last visit or February 1, 2015. CVD interventions considered were ICPs and the use of anti‐hypertensives, angiotensin‐converting enzyme inhibitors (ACEIs) and lipid lowering drugs (LLDs). Individuals with a previous MI/stroke at baseline (i.e. prior to D:A:D Study entry (n = 654)) were excluded from analyses of the subsequent initiation of interventions as the interventions received by these individuals prior to and after the event could not be ascertained with sufficient accuracy. Rates of initiation of each CVD‐related intervention were calculated for the total time of follow‐up and for the specific periods of follow‐up during which individuals were at high CVD risk according to one or more of the following risk subgroups: TC >6.2 mmol/L (>240 mg/dl), TG >2.3 mmol/L (>204 mg/dl), hypertension (systolic blood pressure (SBP) >140 mmHg, diastolic blood pressure (DBP) >90 mmHg, or reported use of ACEIs/anti‐hypertensives), previous (post baseline) MI, diabetes (two consecutive fasting blood glucoses >7.0 mmol/L, HbA1C >6.5% or anti‐diabetic treatment), age >50 years or predicted 10‐year CVD risk score >10% (moderate/high Framingham CVD risk score). As the D:A:D CVD risk score was published in more recent years, the Framingham risk score was chosen as it has been more widely used in participating clinics over the whole study period. Since ACEIs may also be used to treat hypertension, consideration of ACEIs separately to other anti‐hypertensives may result in an under‐estimation of drugs used to treat hypertension. Thus, we additionally considered a combined drug classification of either ACEIs or other anti‐hypertensives.

Each individual's follow‐up was split into a series of consecutive one‐month periods and the clinical, immunologic and virologic status at the start of each period was established. Poisson regression models were then used to assess whether initiation rates of CVD interventions were lower in women compared to men, after adjustment for the following potential time‐updated confounders: age, calendar year, body mass index (BMI), TC, TG, hypertension, previous MI, race, smoking status, AIDS, CVD family history, stroke, diabetes and CVD risk score >10%.

For each calendar year of follow‐up, an individual was considered to have been monitored for TC, TG, HDL and SBP/DBP if there was at least one measure of each within that year. Logistic regression models then assessed whether the probability of being monitored for each measure differed in men and women, after adjustment for calendar year, age, BMI, TC, TG, hypertension, previous MI, diabetes and CVD risk score >10%.

Additional analyses were performed in which we adjusted for TC, TG and SBP/DBP as continuous rather than categorical covariates and after excluding those with a mode of HIV acquisition other than heterosexual sex, as the latter is the group in which the comparison between men and women is least affected by other, unmeasured confounders. Where differences between men and women were identified we fitted a series of regression models, progressively adjusting for each of the potential confounders, allowing us to identify the potential mediators of any differences seen. Finally, since our main analyses investigated overall initiation rates both before and after an MI, we performed sensitivity analyses in which post‐MI follow up was censored, thus restricting analyses to interventions used only prophylactically and allowing us to investigate whether findings were consistent.

## 3 Results

### 3.1 Characteristics at baseline and at time of MI

Of the 49,049 included participants, 12,955 were women and 36,094 were men. Baseline characteristics of the men and women at study entry are shown in Table 1. Most women acquired HIV through heterosexual transmission (9037 (69.8%)), whereas most men acquired HIV through sex with men (21491 (59.5%)). Compared to men, the women were significantly more likely to be younger (median age [interquartile range (IQR)] 34 [29,40] vs. 39 [33,41] years), more likely of black African race (2610 (20.2%) vs. 2220 (6.2%)), less likely to be current smokers (3814 (29.4%) vs. 13556 (37.6%)) or ex‐smokers (1841 (14.2%) vs. 6465 (17.9%)), and less likely to have other traditional CVD risk factors such as diabetes, hypertension, dyslipidemia, previous ICP or receipt of LLDs and ACEIs (Table 1). Furthermore, a higher proportion of women had a low Framingham CVD risk score (<10%), with smaller proportions belonging to the moderate (10‐20%) and high (>20%) CVD risk groups. A slightly higher proportion of women than men had an unknown CVD risk score (Table 1).

**Table 1:** Comparison of characteristics of men and women at D:A:D Study enrolment

		At baseline		*p*‐value^a^
		Men, N (%)	Women, N (%)	
Number		36,094	12,955	
HIV acquisition	MSM	21,491 (59.5)	110 (0.9)	
	IDU	5110 (14.2)	2322 (17.9)	
	Heterosexual	7092 (19.7)	9037 (69.8)	
	Other/not known	2401 (6.7)	1486 (11.5)	0.0001
Race	White	19,017 (52.7)	5795 (44.7)	
	Black African	2220 (6.2)	2610 (20.2)	
	Other	920 (2.6)	484 (3.7)	
	Unknown	13,937 (38.6)	4066 (31.4)	0.0001
Age (years)	Median (IQR)	39 (33, 46)	34 (29, 40)	0.0001
BMI (kg/m^2^)	<18	847 (2.4)	707 (5.5)	
	≥18, ≤26	23,936 (66.3)	7645 (59.0)	
	>26, ≤30	4602 (12.8)	1368 (10.6)	
	>30	1223 (3.4)	914 (7.1)	
	Not known	5486 (15.2)	2321 (17.9)	0.0001
Smoking	Current	13,556 (37.6)	3814 (29.4)	
	Ex‐	6465 (17.9)	1841 (14.2)	
	Never	8186 (22.7)	4800 (37.1)	
	Not known	7887 (21.9)	2500 (19.3)	0.0001
Prior AIDS diagnosis		8285 (23.0)	2769 (21.4)	0.0002
Exposed to ART		21,954 (60.8)	7813 (60.3)	0.30
CD4 (cells/mm^3^)	Median (IQR)	400 (244, 590)	405 (249, 591)	0.06
HIV RNA (log_10_ copies/mL)	Median (IQR)	3.0 (1.7, 4.6)	2.9 (1.7, 4.2)	0.0001
HIV RNA ≤50 copies/mL		10,499 (29.1)	3565 (27.5)	0.0007
Family history of CVD		2325 (6.4)	718 (5.5)	0.0003
Lipodystrophy		5310 (14.7)	1759 (13.6)	0.002
Diabetes		976 (2.7)	221 (1.7)	0.0001
Bypass		26 (0.1)	1 (0.0)	0.007
Endarterectomy		9 (0.0)	1 (0.0)	0.41
Angioplasty		66 (0.2)	3 (0.0)	0.0001
Any ICP		96 (0.3)	5 (0.0)	0.0001
Receipt of LLD		1241 (3.4)	163 (1.3)	0.0001
TC (mmol/L)	Median (IQR)	4.8 (4.0, 5.7)	4.8 (4.0, 5.6)	0.90
TG (mmol/L)	Median (IQR)	1.6 (1.1, 2.7)	1.3 (0.9, 1.9)	0.0001
HDL cholesterol (mmol/L)	Median (IQR)	1.1 (0.8, 1.3)	1.3 (1.1, 1.6)	0.0001
Dyslipidaemia		13831 (38.3)	3049 (23.5)	0.0001
Systolic blood pressure (mmHg)	Median (IQR)	120 (115, 131)	120 (110, 125)	0.0001
Diastolic blood pressure (mmHg)	Median (IQR)	80 (70, 84)	75 (70, 80)	0.0001
Receipt of anti‐hypertensives		1030 (2.9)	349 (2.7)	0.35
Receipt of ACEIs		621 (1.7)	143 (1.1)	0.0001
Hypertension		3904 (10.8)	904 (7.0)	0.0001
Haemoglobin	Median (IQR)	9.0 (8.4, 9.5)	7.9 (7.2, 8.4)	0.0001
eGFR	Median (IQR)	104.2 (89.5, 121.4)	108.8 (88.9, 135.8)	0.0001
Predicted 10‐year CVD risk	Low (<10%)	9879 (27.4)	4148 (32.0)	
	Moderate (10% to 20%)	2378 (6.6)	143 (1.1)	
	High (>20%)	748 (2.1)	22 (0.2)	
	Unknown	23,089 (64.0)	8642 (66.7)	0.0001

MSM, men who have sex with men; IDU, intravenous drug use; BMI, body mass index; ART, anti‐retroviral therapy; CVD, cardiovascular disease; ICPs, invasive cardiovascular procedures; LLDs, lipid lowering drugs; TC, total cholesterol; TG, triglycerides; HDL cholesterol, High‐density lipoprotein cholesterol; ACEIs, Angiotensin‐converting enzyme inhibitors; eGFR, estimated glomerular filtration rate.^a^ *p* < 0.05.

### 3.2 Periods of follow‐up at high CVD risk

The women in the study contributed a total of 113,821 PYRS to the analyses. Of these, 14.9% were contributed by women with a high TG level, 17.5% were contributed by women >50 years, and 16.9% were contributed by women with hypertension (Table 2). The men in the study contributed a total of 314,843 PYRS to the analyses. A higher proportion of this follow‐up time was contributed by men with high TG (29.3%), age >50 years (31.7%) and hypertension (23.8%). Only 4.4% of follow‐up time in women was contributed by women with an established moderate/high CVD risk score (>10%) compared to 27.5% of follow‐up time in men.

**Table 2:** Total duration of follow‐up (person‐years) spent by men and women in one of seven high CVD risk subgroups^a^

High CVD risk group	Men, N (%)	Women, N (%)
Total	314,843 (100.0)	113,821 (100.0)
TC > 6.2 mmol/L (>240 mg/dL)	44,629 (14.2)	15,224 (13.4)
TG > 2.3 mmol/L (>204 mg/dL)	92,397 (29.3)	16,917 (14.9)
Hypertension	75,035 (23.8)	19,195 (16.9)
Previous MI	4206 (1.3)	359 (0.0)
Diabetes	17,226 (5.5)	4170 (3.7)
Age > 50 years	99,911 (31.7)	19,866 (17.5)
CVD risk score >10%	86,425 (27.5)	4990 (4.4)

CVD, cardiovascular disease; TC, total cholesterol; TG, triglycerides; MI, myocardial infarction.

^a^Proportions are not mutually exclusive.

### 3.3 Rates of monitoring for CVD risk factors

Absolute rates of monitoring for TC, TG, HDL‐C and blood pressure within any 12 month period in both low and high CVD risk subgroups of the participants were similar in women and men; TC: 80.3% vs. 81.7%; TG: 76.9 vs. 79.2%; HDL‐C: 64.0 vs 65.3% and blood pressure: 66.6 vs. 65.8%. In unadjusted analyses, women were slightly less likely to be monitored for TC, TG and HDL‐C (TC: odds ratio 0.92 [0.89, 0.96]; TG: 0.88 [0.79, 0.97]; HDL‐C 0.95 [0.88, 1.02]), although differences were attenuated and became non‐significant in adjusted models (TC: 1.01 [0.98, 1.05]; TG: 0.97 [0.93, 1.01]; HDL‐C: 1.03 [0.96, 1.10]). In contrast, while no difference in blood pressure monitoring rates were seen prior to adjustment (0.99 [0.93, 1.05]), women were more likely to be monitored for blood pressure in adjusted models (1.12 [1.08, 1.15]).

### 3.4 Use of CVD interventions

**Figure 1:**
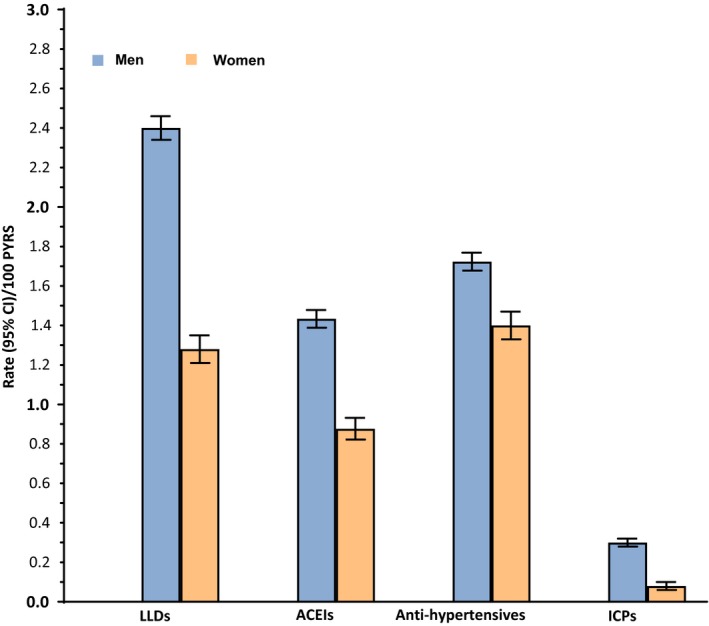
Unadjusted rates of initiation (/100 PYRS) of CVD interventions in women and men, total follow‐up period.

Over the total follow‐up period, 1334 (10.3%) women and 6274 (17.4%) men initiated LLD; 944 (7.3%) women and 4016 (11.1%) men initiated ACEIs; 1444 (11.1%) women and 4834 (13.4%) men initiated anti‐hypertensives; 1715 (13.2%) women and 6126 (17.0%) men initiated ACEIs or anti‐hypertensives; and 89 (0.7%) women and 932 (2.6%) men underwent an ICP. When taking all follow‐up time into consideration, women had lower initiation rates than men for all CVD interventions: LLDs (incidence rate (IR) [95% CI]/100 PYRS in women vs. men 1.28 [1.21, 1.35] vs. 2.40 [2.34, 2.46]), ACEIs (0.88 [0.82, 0.93] vs. 1.43 [1.39, 1.48]), anti‐hypertensives (1.40 [1.33, 1.47] vs. 1.72 [1.68, 1.77]); ACEIs or anti‐hypertensives 1.59 [1.61, 1.77] vs. 2.26 [2.21, 2.32] and ICPs (0.08 [0.06, 0.10] vs. 0.30 [0.28, 0.32]) (Figure 1).

When restricting the analysis only to periods of follow‐up during which an individual was in one of the high CVD risk subgroups, women generally continued to have lower initiation rates than men for most CVD interventions (Table 3). The only exceptions to this were for the receipt of LLDs, ACEIs, anti‐hypertensives and ACEIs or anti‐hypertensives in people with a CVD risk score >10%; the uptake of anti‐hypertensives and ACEIs or anti‐hypertensives among people with hypertension and the receipt of ACEIs among people with a previous MI. In each of these latter subgroups, initiation rates of the interventions were higher in women than men (Table 3).

**Table 3:** Rates of initiating LLD, ACEIs, anti‐hypertensive drugs and ICPs in men and women in high risk subgroups of the population

Risk subgroup		LLD		ACEIs		Anti‐hypertensives		ICPs		Anti‐hypertensives OR ACEIs	
		No. initiating/PYRS	Rate (95% CI)/100 PYRS	No. initiating/PYRS	Rate (95% CI)/100 PYRS	No. initiating/PYRS	Rate (95% CI)/100 PYRS	No. initiating/PYRS	Rate (95% CI)/100 PYRS	No. initiating/PYRS	Rate (95% CI)/100 PYRS
High TC	M	3045/31,383	9.70 (9.36, 10.05)	854/40,141	2.13 (1.99, 2.27)	961/39,105	2.46 (2.30, 2.61)	275/44,118	0.62 (0.55, 0.70)	1263/37,343	3.38 (3.20, 3.57)
	F	764/11,948	6.39 (594, 6.85)	206/13,971	1.47 (1.27, 1.68)	241/13,275	1.82 (1.59, 2.05)	24/15,172	0.16 (0.10, 0.22)	306/12,869	2.38 (2.11, 2.64)
High TG	M	3533/66,036	5.35 (5.17, 5.53)	1599/81,811	1.96 (1.86, 2.05)	1760/79,319	2.22 (2.12, 2.32)	452/90,461	0.50 (0.45, 0.55)	2287/75,551	3.03 (2.910, 3.15)
	F	539/13,511	3.99 (3.,65, 4.33)	216/15,508	1.39 (1.21, 1.58)	284/14,784	1.92 (1.70, 2.14)	28/16,811	0.17 (0.11, 0.23)	344/14,310	2.40 (2.15, 2.66)
Hyper‐tension	M	2078/49,162	4.23 (4.05, 4.41)	2598/48,266	5.38 (5.18, 5.59)	2390/40,624	5.88 (5.65, 6.12)	489/70,760	0.69 (0.63, 0.75)	2729/30,875	8.84 (8.51, 9.17)
	F	414/15,005	2.76 (2.49, 3.03)	597/13,305	4.49 (4.13, 4.85)	592/8662	6.83 (6.28, 7.39)	43/18,836	0.23 (0.16, 0.30)	618/6765	9.14 (8.42, 9.86)
Previous MI	M	143/671	21.31 (17.82, 24.80)	144/1758	8.19 (6.85, 9.53)	157/858	18.30 (15.44, 21.16)	97/1045	9.28 (7.44, 11.13)	149/701	21.26 (17.84, 24.67)
	F	16/108	14.82 (7.56, 22.07)	18/169	10.65 (6.31, 16.83)	15/93	16.13 (9.03, 24.29)	5/94	5.32 (1.73, 12.42)	12/87	13.79 (7.13, 24.09)
Diabetes	M	650/9059	7.18 (6.62, 7.73)	568/11,966	4.75 (4.36, 5.14)	532/11,535	4.61 (4.22, 5.00)	148/16,297	0.91 (0.76, 1.05)	680/9701	7.01 (6.48, 7.54)
	F	132/2757	4.79 (3.97, 5.61)	101/3212	3.14 (2.53, 3.76)	108/2890	3.74 (3.03, 4.44)	13/4117	0.32 (0.17, 0.54)	140/2583	5.42 (4.52, 6.32)
Over 50 years	M	2921/69,055	4.23 (4.08, 4.38)	2216/82,105	2.70 (2.59, 2.81)	2523/77,655	3.25 (3.12, 3.38)	594/96,327	0.62 (0.57, 0.67)	3147/71,696	4.39 (4.24, 4.54)
	F	527/15,055	3.50 (3.20, 3.80)	377/16,904	2.23 (2.01, 2.46)	442/15,366	2.88 (2.61, 3.15)	42/19,627	0.21 (0.15, 0.28)	546/14,438	3.78 (3.46, 4.10)
Moderate/high 10‐year CVD risk	M	3310/55,991	5.91 (5.71, 6.11)	2263/69,858	3.24 (3.11, 3.37)	2372/66,247	3.58 (3.44, 3.73)	636/82,088	0.78 (0.72, 0.84)	3029/60,804	4.98 (4.80, 5.16)
	F	242/2993	8.09 (7.07, 9.10)	164/3787	4.33 (3.67, 4.99)	167/3358	4.97 (4.22, 5.73)	27/4698	0.58 (0.36, 0.79)	199/3028	6.57 (5.66, 7.49)

M, male; F, female; LLDs, lipid lowering drugs; ACEIs, Angiotensin‐converting enzyme inhibitors; ICPs, invasive cardiovascular procedures; TC, total cholesterol; TG, triglycerides; MI, myocardial infarction; CVD, cardiovascular disease.

### 3.5 Poisson regression models

**Figure 2:**
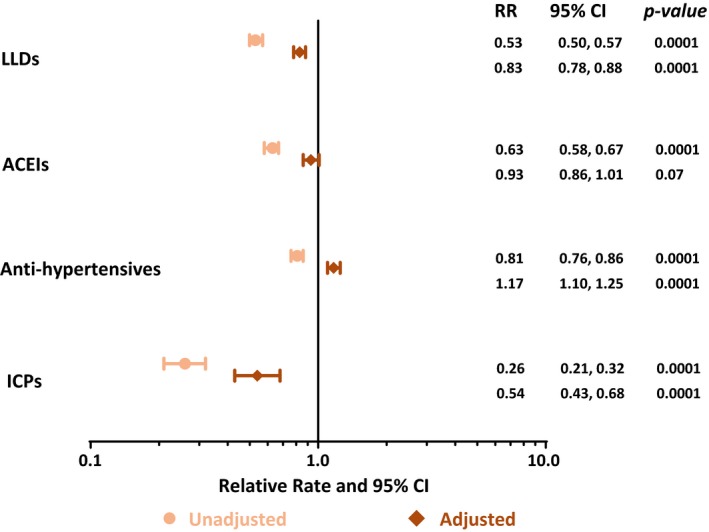
Relative rates of receipt of CVD interventions in women compared to men. Multivariate model adjusted for: Age, calendar year, body mass index, total cholesterol, triglycerides, hypertension, previous myocardial infarction, race, smoking status, AIDS, cardiovascular disease (CVD) family history, stroke, diabetes and CVD risk score >10% (individuals having a moderate or high Framingham CVD risk score). CVD, cardiovascular disease; RR, relative rate; 95% CI, 95% confidence interval; LLDs, lipid lowering drugs; ACEIs, Angiotensin‐converting enzyme inhibitors; ICPs, invasive cardiovascular procedures. *p < 0.05.

In Poisson regression models, unadjusted rates of initiation for each of the four CVD interventions (LLDs, ACEIs, anti‐hypertensives and ICPs) were lower in women than in men (Figure 2). When adjusting for potential confounders, rate ratios were attenuated but still indicated significantly lower initiation rates in women compared to men for LLDs and ICPs: LLDs: (relative rate (RR)) 0.83 [0.78, 0.88]; ICPs: 0.54 [0.43, 0.68], and borderline significantly lower rates for ACEIs (0.93 [0.86, 1.01]). For anti‐hypertensives, the direction of the association was reversed after adjustment for potential confounders, reflecting a higher initiation rate in women compared to men (1.17 [1.10, 1.25]) (Figure 2). This was also observed when we considered initiation of either anti‐hypertensives *or* ACEIs (1.08 [1.02, 1.15]). To investigate which factors were likely to contribute to the higher likelihood of use of anti‐hypertensives by women compared to men, a series of regression models was fitted in which we progressively adjusted for each of the potential confounders in turn. This analysis revealed that this finding was mainly driven by adjustments for hypertension and a CVD risk score >10%.

Additional adjustment for TC, TG, and SBP/DBP as continuous covariates, and the exclusion of those with modes of HIV transmission other than heterosexual sex, led to consistent results. Consistent results were also observed when follow‐up was censored at the time of an MI, suggesting that our findings could not simply be explained by a higher uptake of secondary prevention interventions post‐MI in men.

## 4 Discussion

Women generally have a lower CVD risk than men, particularly at younger ages 10, 11, 12, 13, 14, 15, 16. While an increased risk of CVD in HIV‐positive individuals is well recognized 1, 2, 3, 4, 5, 6, 7, 8, this increased risk has been noted to be more pronounced in HIV‐positive women than in HIV‐positive men 2, 3, 4, 8, 9. To the best of our knowledge, however, this is the first study to specifically investigate gender differences in the management of CVD between HIV‐positive women and men, with substantial follow‐up time and rigorously monitored and centrally validated events and interventions. In our study, we observed that women had a lower overall CVD risk at baseline, that blood pressure was more likely to be monitored in women, and that initiation rates of CVD interventions were generally lower in women than in men. This was also true for most CVD interventions when analyses were restricted to periods of follow‐up during which each person was at high CVD risk. In fully adjusted models, women were less likely than men to receive LLDs, ACEIs and ICPs, although conversely were more likely to receive anti‐hypertensives.

We identified subgroups of study participants who we believed would be considered to be at higher CVD risk and in whom monitoring and interventions for CVD might be appropriate. Where women were deemed to be at high CVD risk, this was most commonly due to the presence of hypertension and/or triglyceridemia, or because of older age. The relatively high proportion of time that women spent with hypertension likely reflects the higher proportion of those of black African ethnicity, a known risk factor for hypertension 41, among women than men. When restricting the analysis only to periods of follow‐up during which an individual was in one of the high CVD risk subgroups, women generally had lower initiation rates than men for most CVD interventions. Among younger, pre‐menopausal women, it would not be surprising to see lower initiation rates, reflecting the lower overall CVD risk. However, as women age, their CVD risk becomes more similar to that of men 10, 11, 12, 13, 14, 15, 16 and thus we might have expected more similar initiation rates of CVD interventions in the older age group. While only a relatively low proportion of follow‐up time among women was contributed by those with a CVD risk score >10%, a higher proportion of follow‐up time in women could not be categorized due to missing data on one or more components of the score. Hence, this “high‐risk” category may miss women with a genuinely high risk who had not been identified as such. Although the initiation rates of LLDs, ACEIs and anti‐hypertensives for women with a CVD risk score >10% were higher in women than for men, we do not think that we can focus solely on this high risk group, as the other separate high CVD risk indicators should also prompt concern about high CVD risk. Furthermore, these unadjusted rates did not take into account differences in other characteristics.

In fully adjusted models, women in our study were less likely than men to receive LLDs, ACEIs and ICPs, and of these interventions least likely to receive ICPs. Several other studies from the general population have also demonstrated that women are less likely to receive diagnostic and therapeutic invasive CVD‐interventions than men 11, 12, 16, 20, 30, 31, 32, 33, 34, and that women have higher mortality after invasive procedures 16, 27, 28, 29. Women are less likely to have ST‐elevation myocardial infarction and more likely to have either none or atypical symptoms at the time of MI 42, 43, 44, 45, 46, possibly partly explained by female‐specific characteristics in macro‐and microvasculature 47, 48, 49. For stroke, women tend to present with more generalized symptoms such as dizziness, headache, disorientation and changes in consciousness as well as other atypical symptoms compared to men 16. These more subtle and heterogeneous clinical presentations, in addition to potential gender‐related differences in diagnostic biomarkers and social factors, may complicate and/or delay the diagnosis and management of MI and stroke in women 16, 32, 33, 42, 50, 51, 52, 53. Furthermore, at the time of MI and stroke, women also tend to be older, to have more severe CVD risk profiles and more comorbidities 10, 11, 12, 13, 14, 15, 16, 42, 54 than men, which may also complicate interventions and contribute to a poorer prognosis. Finally, lesser use of invasive CVD‐interventions in women may also be due to differences in angiographic features; women are not always eligible for treatment with stents or grafts due to microvascular rather than obstructive coronary disease, as well as lesser degree of carotid stenosis which is less suitable for stenting 16, 31, 49.

In contrast to the receipt of LLDs, ACEIs and ICPs, women in our study were more likely to receive anti‐hypertensives than men. The increased use of anti‐hypertensives appeared to be driven by women with hypertension and a CVD risk score >10%. As ACEIs are partly used as anti‐hypertensives, the gender difference in the use of ACEIs was expectedly reduced after controlling for hypertension, and the difference between men and women was less marked when the combined class of anti‐hypertensives or ACEIs was considered, with the lower uptake in women for ACEIs being counter‐balanced by the higher uptake of anti‐hypertensives in this group. Supported by the slightly higher monitoring rates for blood pressure that we observed in women, our findings argue that hypertension might have more focus in clinical practice. This may relate to hypertension being one of the more common CVD risk factors in women 42, 54, to increased monitoring among pregnant women and women on contraceptives, as well as the relatively large proportion of women being of black African origin.

### 4.1 Limitations

While we capture information on many CVD risk factors, some CVD risk factors and preventive CVD‐interventions (e.g. smoking cessation in smokers, advice on diet, exercise and the use of over‐the‐counter drugs such as aspirin) as well as some female‐specific factors (e.g. pregnancy, menopausal status, hormone supplementation and contraceptives) are not captured in our dataset. For this reason, we did not aim to identify specific individuals in whom interventions would be certain to be recommended or to assess whether any such recommendations were appropriate, but simply used the data to identify groups at higher CVD risk in whom awareness of CVD risk and regular monitoring should be greater. It is possible that some individuals may not have accepted any interventions they were offered, or that provider or health system‐related factors, (e.g. availability of specialized cardiac care), may have influenced our results, possibilities we are unable to investigate due to the nature of the dataset. Although we believe that our detailed query processes and monitoring activities contribute to minimize ascertainment bias, we cannot exclude the possibility that there may be under‐ or delayed‐ascertainment of the receipt of CVD interventions or that this information may be less readily available in women than men. A relatively small proportion of follow‐up time among women was contributed by those at moderate or high CVD risk, but still represented around 5000 PYRS, and our results suggest that the study is not under‐powered to detect effects. Finally, although the differences observed are intriguing, we are unable to investigate the reasons for these gender differences.

## 5 Conclusion

In our study, HIV‐positive women were less likely than men to receive most CVD‐related interventions, with the exception of anti‐hypertensive drugs. These findings are mostly consistent with those from the general population.

The reasons why women are less likely to receive interventions than men are multiple, but insufficient monitoring and awareness of CVD risk in women, and the more heterogeneous clinical presentations of CVD probably play a major role. As HIV‐positive individuals in general are at higher risk of CVD further efforts are needed to ensure that both women and men are appropriately monitored for CVD risk and, if required, receive relevant CVD‐related interventions. Furthermore studies are warranted on why these gender related differences exist in the prevention and management of CVD in HIV‐positive individuals.

## Competing interests

Amanda Mocroft has received travel support, honoraria, speaker fees and/or lecture fees from BMS, Gilead, ViiV, Pfizer, Merck, BI and Wragge LLC.

Peter Reiss has through his institution received independent scientific grant support from Gilead Sciences, Janssen Pharmaceuticals Inc, Merck & Co, Bristol‐Myers Squibb and ViiV Healthcare; he has served on a scientific advisory board for Gilead Sciences and a data safety monitoring committee for Janssen Pharmaceuticals Inc; he chaired a scientific symposium by ViiV Healthcare, for which his institution has received remuneration.

Christian Pradier reports non‐financial support from JANSSEN, personal fees from GILEAD, non‐financial support from VIIV HEALTH CARE, non‐financial support from MSD, outside the submitted work.

Antonella d'Arminio Monforte has received grants for advisory boards or lectures by Abbve, BMS, Gilead, Janssen, MSD, ViiV

Matthew Law has received unrestricted grants from Boehringer Ingelhiem, Gilead Sciences, Merck Sharp & Dohme, Bristol‐Myers Squibb, Janssen‐Cilag, ViiV HealthCare. Consultancy payments from Gilead Sciences DSMB sitting fees from Sirtex Pty Ltd

Caroline Sabin has received honoraria for the membership of Data Safety and Monitoring Boards, Advisory Boards and Speaker Panels from Gilead Sciences, ViiV Healthcare and Janssen‐Cilag. She has received funding to support the development of educational materials from Gilead Sciences and ViiV Healthcare.

Camilla Ingrid Hatleberg, Lene Ryom, Wafaa El‐Sadr, Helen Kovari, Francois Dabis, Stephane de Wit and Jens Lundgren have no disclosures to declare.

## Authors' contributions

Author contributions: C.I.H, L.R., J.D.L. and C.S. developed the initial analysis protocol. C.I.H and L.R performed study co‐ordination and prepared the datasets for analysis, C.S. performed the statistical analysis. C.I.H. prepared the first draft of the manuscript and completed all revisions. L.R, J.D.L and C.S provided critical input at all stages of the preparation of the manuscript. W.E.S, A.M, P.R, S.D.W, F.D, C.P, A.D.M, H.K, M. L provided data and revised the manuscript critically. All authors have provided input at all stages of the project and approved the final version.

## Funding Information

Grant number DNRF126 from the Danish National Research Foundation (CHIP & PERSIMUNE); “Oversight Committee for The Evaluation of Metabolic Complications of HAART” with representatives from academia, patient community, FDA, EMA and a consortium of AbbVie, Bristol‐Myers Squibb, Gilead Sciences, ViiV Healthcare, Merck and Janssen Pharmaceuticals.

### The current members of the 11 Cohorts are as follows

#### ATHENA (AIDS Therapy Evaluation Project Netherlands)

Central coordination: P Reiss*, S Zaheri, M Hillebregt, FWNM Wit;

CLINICAL CENTRES (¤ denotes site coordinating physician) Academic Medical Centre of the University of Amsterdam: JM Prins¤, TW Kuijpers, HJ Scherpbier, JTM van der Meer, FWNM Wit, MH Godfried, P Reiss,T van der Poll, FJB Nellen, SE Geerlings, M van Vugt, D Pajkrt, JC Bos, WJ Wiersinga, M van der Valk, A Goorhuis, JW Hovius, J van Eden, A Henderiks, AMH van Hes, M Mutschelknauss, HE Nobel, FJJ Pijnappel, S Jurriaans, NKT Back, HL Zaaijer, B Berkhout, MTE Cornelissen, CJ Schinkel, XV Thomas. A De Ruyter Ziekenhuis, Goes: M van den Berge, A Stegeman, S Baas, L Hage de Looff, D Versteeg. C Ziekenhuis, Eindhoven: MJH Pronk¤, HSM Ammerlaan, E de Munnik . AR Jansz, J Tjhie, MCA Wegdam, B Deiman, V Scharnhorst. Emma Kinderziekenhuis: A van der Plas, AM Weijsenfeld. Erasmus MC, Rotterdam: ME van der Ende¤, TEMS de Vries‐Sluijs, ECM van Gorp, CAM Schurink, JL Nouwen, A Verbon, BJA Rijnders, HI Bax, M van der Feltz, N Bassant, JEA van Beek, M Vriesde, LM van Zonneveld. A de Oude‐Lubbers, HJ van den Berg‐Cameron, FB Bruinsma‐Broekman, J de Groot, M de Zeeuw‐de Man, CAB Boucher, MPG Koopmans, JJA van Kampen, SD Pas. Erasmus MC–Sophia, Rotterdam: GJA Driessen, AMC van Rossum, LC van der Knaap, E Visser Flevoziekenhuis, Almere: J Branger¤, A Rijkeboer‐Mes, CJHM Duijf‐van de Ven. HagaZiekenhuis, Den Haag: EF Schippers¤, C van Nieuwkoop, JM van IJperen, J Geilings, G van der Hut, PFH Franck. HIV Focus Centrum (DC Klinieken): A van Eeden¤, W Brokking, M Groot, LJM Elsenburg, M Damen, IS Kwa Isala, Zwolle: PHP Groeneveld¤, JW Bouwhuis, JF van den Berg, AGW van Hulzen, GL van der Bliek, PCJ Bor, P Bloembergen, MJHM Wolfhagen, GJHM Ruijs. Leids Universitair Medisch Centrum, Leiden: FP Kroon¤, MGJ de Boer, MP Bauer, H Jolink, AM Vollaard, W Dorama, N van Holten, ECJ Claas, E Wessels. Maasstad Ziekenhuis, Rotterdam: JG den Hollander¤, K Pogany, A Roukens, M Kastelijns, JV Smit, E Smit, D Struik‐Kalkman, C Tearno, M Bezemer, T van Niekerk, O Pontesilli. Maastricht UMC+, Maastricht: SH Lowe¤, AML Oude Lashof, D Posthouwer, RP Ackens, J Schippers, R Vergoossen, B Weijenberg‐Maes, IHM van Loo, TRA Havenith. MCH‐Bronovo, Den Haag: EMS Leyten¤, LBS Gelinck, A van Hartingsveld, C Meerkerk, GS Wildenbeest, JAEM Mutsaers, CL Jansen. MC Slotervaart, Amsterdam: JW Mulder, SME Vrouenraets, FN Lauw, MC van Broekhuizen, H Paap, DJ Vlasblom, PHM Smits. MC Zuiderzee, Lelystad: S Weijer¤, R El Moussaoui, AS Bosma. Medisch Centrum Leeuwarden, Leeuwarden: MGA van Vonderen¤, DPF van Houte, LM Kampschreur, K Dijkstra, S Faber, J Weel. Medisch Spectrum Twente, Enschede: GJ Kootstra¤, CE Delsing, M van der Burg‐van de Plas, H Heins, E Lucas. Noorwest Ziekenhuisgroep, Alkmaar: W Kortmann¤, G van Twillert¤, JWT Cohen Stuart, BMW Diederen, D Pronk, FA van Truijen‐Oud, WA van der Reijden, R Jansen. OLVG, Amsterdam: K Brinkman¤, GEL van den Berk, WL Blok, PHJ Frissen, KD Lettinga, WEM Schouten, J Veenstra, CJ Brouwer, GF Geerders, K Hoeksema, MJ Kleene, IB van der Meché, M Spelbrink, H Sulman, AJM Toonen, S Wijnands, M Damen, D Kwa, E Witte. Radboudumc, Nijmegen: PP Koopmans, M Keuter, AJAM van der Ven, HJM ter Hofstede, ASM Dofferhoff, R van Crevel, M Albers, MEW Bosch, KJT Grintjes‐Huisman, BJ Zomer, FF Stelma, J Rahamat‐Langendoen, D Burger. Rijnstate, Arnhem: C Richter¤, EH Gisolf, RJ Hassing, G ter Beest, PHM van Bentum, N Langebeek, R Tiemessen, CMA Swanink. Spaarne Gasthuis, Haarlem: SFL van Lelyveld¤, R Soetekouw, N Hulshoff, LMM van der Prijt, J van der Swaluw, N Bermon, WA van der Reijden, R Jansen, BL Herpers, D Veenendaal. Medisch Centrum Jan van Goyen, Amsterdam: DWM Verhagen, M van Wijk. St Elisabeth Ziekenhuis, Tilburg: MEE van Kasteren¤, AE Brouwer, BAFM de Kruijf‐van de Wiel, M Kuipers, RMWJ Santegoets, B van der Ven, JH Marcelis, AGM Buiting, PJ Kabel. Universitair Medisch Centrum Groningen, Groningen: WFW Bierman¤, H Scholvinck, KR Wilting, Y Stienstra, H de Groot‐de Jonge, PA van der Meulen, DA de Weerd, J Ludwig‐Roukema, HGM Niesters, A Riezebos‐Brilman, CC van Leer‐Buter, M Knoester. Universitair Medisch Centrum Utrecht, Utrecht: AIM Hoepelman¤, T Mudrikova, PM Ellerbroek, JJ Oosterheert, JE Arends, RE Barth, MWM Wassenberg, EM Schadd, DHM van Elst‐Laurijssen, EEB van Oers‐Hazelzet, S Vervoort, M van Berkel, R Schuurman, F Verduyn‐Lunel, AMJ Wensing. VUmc, Amsterdam: EJG Peters¤, MA van Agtmael, M Bomers, J de Vocht, M Heitmuller, LM Laan, AM Pettersson, CMJE Vandenbroucke‐Grauls, CW Ang . Wilhelmina Kinderziekenhuis, UMCU, Utrecht: SPM Geelen, TFW Wolfs, LJ Bont, N Nauta. COORDINATING CENTRE P Reiss, DO Bezemer, AI van Sighem, C Smit, FWNM Wit, TS Boender, S Zaheri, M Hillebregt, A de Jong, D Bergsma, P Hoekstra, A de Lang, S Grivell, A Jansen, MJ Rademaker, M Raethke, R Meijering, S Schnörr, L de Groot, M van den Akker, Y Bakker, E Claessen, A El Berkaoui, J Koops, E Kruijne, C Lodewijk, L Munjishvili, B Peeck, C Ree, R Regtop, Y Ruijs, T Rutkens, L van de Sande, M Schoorl, A Timmerman, E Tuijn, L Veenenberg, S van der Vliet, A Wisse, T Woudstra, B Tuk.

### Aquitaine Cohort (France)

#### Composition du Conseil scientifique

Coordination: F Bonnet, F Dabis

Scientific committee: M Dupon, V Gaborieau, D Lacoste, D Malvy, P Mercié, P Morlat, D Neau, JL Pellegrin, S Tchamgoué, E Lazaro, C Cazanave, M Vandenhende, MO Vareil, Y Gérard, P Blanco, S Bouchet, D Breilh, H Fleury, I Pellegrin, G Chêne, R Thiébaut, L Wittkop, L Wittkop, O Leleux, S Lawson‐Ayayi, A Gimbert, S Desjardin, L Lacaze‐Buzy, V Petrov‐Sanchez

Epidemiology and Methodology: F Bonnet, G Chêne, F Dabis, R Thiébaut, L Wittkop

Infectious Diseases and Internal Medicine: K André, N Bernard, F Bonnet, O Caubet, L Caunegre, C Cazanave, I Chossat, C Courtault, FA Dauchy, S De Witte, D Dondia, M Dupon, P Duffau, H Dutronc, S Farbos, I Faure, H Ferrand, V Gaborieau, Y Gerard, C Greib, M Hessamfar, Y Imbert, D Lacoste, P Lataste, E Lazaro, D Malvy, J Marie, M Mechain, P Mercié, E Monlun, P Morlat, D Neau, A Ochoa, JL Pellegrin, T Pistone, I Raymond, MC Receveur, P Rispal, L Sorin, S Tchamgoué, C Valette, MA Vandenhende, MO Vareil, JF Viallard, H Wille, G Wirth.

Immunology: I Pellegrin, P Blanco

Virology: H Fleury, ME Lafon, P Trimoulet, P Bellecave, C Tumiotto

Pharmacology: S Bouchet, D Breilh, F Haramburu, G Miremeont‐Salamé

Data collection, Project Management and Statistical Analyses: MJ Blaizeau, M Decoin, C Hannapier, E Lenaud et A Pougetoux; S Delveaux, C D'Ivernois, F Diarra, B Uwamaliya‐Nziyumvira, O Leleux; F Le Marec, E Boerg, S Lawson‐Ayayi;

IT department and eCRF development: G Palmer, V Conte, V Sapparrart

### AHOD (Australian HIV Observational Database, Australia)

Central coordination: M Law*, K Petoumenos, R Puhr, R Huang (Sydney, New South Wales). Participating physicians (city, state): R Moore, S Edwards, J Hoy, K Watson, N Roth, H Lau (Melbourne, Victoria); M Bloch, D Baker, A Carr, D Cooper, (Sydney, New South Wales); M O'Sullivan (Gold Coast, Queensland), D Nolan, G Guelfi (Perth, Western Australia).

### BASS (Spain)

Central coordination: G Calvo, F Torres, S Mateu (Barcelona);

Participating physicians (city): P Domingo, MA Sambeat, J Gatell, E Del Cacho, J Cadafalch, M Fuster (Barcelona); C Codina, G Sirera, A Vaqué (Badalona).

### The Brussels St Pierre Cohort (Belgium)

Coordination: S De Wit*, N Clumeck, M Delforge, C Necsoi.

Participating physicians: N Clumeck, S De Wit*, AF Gennotte, M Gerard, K Kabeya, D Konopnicki, A Libois, C Martin, MC Payen, P Semaille, Y Van Laethem.

### CPCRA (USA)

Central coordination: J Neaton, G Bartsch, WM El‐Sadr*, E Krum, G Thompson, D Wentworth;

Participating physicians (city, state): R Luskin‐Hawk (Chicago, Illinois); E Telzak (Bronx, New York); WM El‐Sadr (Harlem, New York); DI Abrams (San Francisco, California); D Cohn (Denver, Colorado); N Markowitz (Detroit, Michigan); R Arduino (Houston, Texas); D Mushatt (New Orleans, Louisiana); G Friedland (New Haven, Connecticut); G Perez (Newark, New Jersey); E Tedaldi (Philadelphia, Pennsylvania); E Fisher (Richmond, Virginia); F Gordin (Washington, DC); LR Crane (Detroit, Michigan); J Sampson (Portland, Oregon); J Baxter (Camden, New Jersey).

### EuroSIDA (multinational)

Steering Committee: J Gatell, B Gazzard, A Horban, I Karpov, M Losso, A d'Arminio Monforte, C Pedersen, M Ristola, A Phillips, P Reiss, J Lundgren, J Rockstroh

Chair: J Rockstroh

Study Co‐leads: A Mocroft, O Kirk

Coordinating Centre Staff: O Kirk, L Peters, C Matthews, AH Fischer, A Bojesen, D Raben, D Kristensen, K Grønborg Laut, JF Larsen, D Podlekareva

Statistical Staff: A Mocroft, A Phillips, A Cozzi‐Lepri, L Shepherd, A Schultze, S Amele

The multi‐centre study group, EuroSIDA (national coordinators in parenthesis).

Argentina: (M Losso), M Kundro, Hospital JM Ramos Mejia, Buenos Aires.

Austria: (B Schmied), Pulmologisches Zentrum der Stadt Wien, Vienna; R Zangerle, Medical University Innsbruck, Innsbruck.

Belarus: (I Karpov), A Vassilenko, Belarus State Medical University, Minsk, VM Mitsura, Gomel State Medical University, Gomel; D Paduto, Regional AIDS Centre, Svetlogorsk.

Belgium: (N Clumeck), S De Wit, M Delforge, Saint‐Pierre Hospital, Brussels; E Florence, Institute of Tropical Medicine, Antwerp; L Vandekerckhove, University Ziekenhuis Gent, Gent.

Bosnia‐Herzegovina: (V Hadziosmanovic), Klinicki Centar Univerziteta Sarajevo, Sarajevo.

Croatia: (J Begovac), University Hospital of Infectious Diseases, Zagreb.

Czech Republic: (L Machala), D Jilich, Faculty Hospital Bulovka, Prague; D Sedlacek, Charles University Hospital, Plzen.

Denmark: G Kronborg, T Benfield, Hvidovre Hospital, Copenhagen; J Gerstoft, T Katzenstein, Rigshospitalet, Copenhagen; NF Møller, C Pedersen, Odense University Hospital, Odense; L Ostergaard, Skejby Hospital, Aarhus, L Wiese, Roskilde Hospital, Roskilde; LN Nielsen, Hillerod Hospital, Hillerod.

Estonia: (K Zilmer), West‐Tallinn Central Hospital, Tallinn; Jelena Smidt, Nakkusosakond Siseklinik, Kohtla‐Järve.

Finland: (M Ristola), I Aho, Helsinki University Central Hospital, Helsinki.

France: (J‐P Viard), Hôtel‐Dieu, Paris; P‐M Girard, Hospital Saint‐Antoine, Paris; C Pradier, E Fontas, Hôpital de l'Archet, Nice; C Duvivier, Hôpital Necker‐Enfants Malades, Paris.

Germany: (J Rockstroh), Universitäts Klinik Bonn; R Schmidt, Medizinische Hochschule Hannover; O Degen, University Medical Center Hamburg‐Eppendorf, Infectious Diseases Unit, Hamburg; HJ Stellbrink, IPM Study Center, Hamburg; C Stefan, JW Goethe University Hospital, Frankfurt; J Bogner, Medizinische Poliklinik, Munich; G Fätkenheuer, Universität Köln, Cologne.

Georgia: (N Chkhartishvili) Infectious Diseases, AIDS & Clinical Immunology Research Center, Tbilisi

Greece: (P Gargalianos), G Xylomenos, K Armenis, Athens General Hospital “G Gennimatas”; H Sambatakou, Ippokration General Hospital, Athens.

Hungary: (J Szlávik), Szent Lásló Hospital, Budapest.

Iceland: (M Gottfredsson), Landspitali University Hospital, Reykjavik.

Ireland: (F Mulcahy), St. James's Hospital, Dublin.

Israel: (I Yust), D Turner, M Burke, Ichilov Hospital, Tel Aviv; E Shahar, G Hassoun, Rambam Medical Center, Haifa; H Elinav, M Haouzi, Hadassah University Hospital, Jerusalem; D Elbirt, ZM Sthoeger, AIDS Center (Neve Or), Jerusalem.

Italy: (A d'Arminio Monforte), Istituto Di Clinica Malattie Infettive e Tropicale, Milan; R Esposito, I Mazeu, C Mussini, Università Modena, Modena; F Mazzotta, A Gabbuti, Ospedale S Maria Annunziata, Firenze; V Vullo, M Lichtner, University di Roma la Sapienza, Rome; M Zaccarelli, A Antinori, R Acinapura, M Plazzi, Istituto Nazionale Malattie Infettive Lazzaro Spallanzani, Rome; A Lazzarin, A Castagna, N Gianotti, Ospedale San Raffaele, Milan; M Galli, A Ridolfo, Osp. L. Sacco, Milan.

Latvia: (B Rozentale), Infectology Centre of Latvia, Riga.

Lithuania: (V Uzdaviniene) Vilnius University Hospital Santariskiu Klinikos, Vilnius; R Matulionyte, Center of Infectious Diseases, Vilnius University Hospital Santariskiu Klinikos, Vilnius.

Luxembourg: (T Staub), R Hemmer, Centre Hospitalier, Luxembourg.

Netherlands: (P Reiss), Academisch Medisch Centrum bij de Universiteit van Amsterdam, Amsterdam.

Norway: (V Ormaasen), A Maeland, J Bruun, Ullevål Hospital, Oslo.

Poland: (B Knysz), J Gasiorowski, M Inglot, Medical University, Wroclaw; A Horban, E Bakowska, Centrum Diagnostyki i Terapii AIDS, Warsaw; R Flisiak, A Grzeszczuk, Medical University, Bialystok; M Parczewski, K Maciejewska, B Aksak‐Was, Medical Univesity, Szczecin; M Beniowski, E Mularska, Osrodek Diagnostyki i Terapii AIDS, Chorzow; T Smiatacz, M Gensing, Medical University, Gdansk; E Jablonowska, E Malolepsza, K Wojcik, Wojewodzki Szpital Specjalistyczny, Lodz; I Mozer‐Lisewska, Poznan University of Medical Sciences, Poznan.

Portugal: (L Caldeira), Hospital Santa Maria, Lisbon; K Mansinho, Hospital de Egas Moniz, Lisbon; F Maltez, Hospital Curry Cabral, Lisbon.

Romania: (R Radoi), C Oprea, Spitalul de Boli Infectioase si Tropicale: Dr. Victor Babes, Bucarest.

Russia: (A Panteleev), O Panteleev, St Petersburg AIDS Centre, St Peterburg; A Yakovlev, Medical Academy Botkin Hospital, St Petersburg; T Trofimora, Novgorod Centre for AIDS, Novgorod, I Khromova, Centre for HIV/AIDS & and Infectious Diseases, Kaliningrad; E Kuzovatova, Nizhny Novgorod Scientific and Research Institute of Epidemiology and Microbiology named after Academician I.N. Blokhina, Nizhny Novogrod; E Borodulina, E Vdoushkina, Samara State Medical University, Samara.

Serbia: (D Jevtovic), The Institute for Infectious and Tropical Diseases, Belgrade.

Slovenia: (J Tomazic), University Clinical Centre Ljubljana, Ljubljana.

Spain: (JM Gatell), JM Miró, Hospital Clinic Universitari de Barcelona, Barcelona; S Moreno, JM Rodriguez, Hospital Ramon y Cajal, Madrid; B Clotet, A Jou, R Paredes, C Tural, J Puig, I Bravo, Hospital Germans Trias i Pujol, Badalona; P Domingo, M Gutierrez, G Mateo, M Sambeat, Hospital Sant Pau, Barcelona; JM Laporte, Hospital Universitario de Alava, Vitoria‐Gasteiz.

Sweden: (K Falconer), A Thalme, A Sonnerborg, Karolinska University Hospital, Stockholm; A Blaxhult, Venhälsan‐Sodersjukhuset, Stockholm; L Flamholc, Malmö University Hospital, Malmö.

Switzerland: (A Scherrer), R Weber, University Hospital Zurich; M Cavassini, University Hospital Lausanne; A Calmy, University Hospital Geneva; H Furrer, University Hospital Bern; M Battegay, University Hospital Basel; P Schmid, Cantonal Hospital St. Gallen.

Ukraine: A Kuznetsova, Kharkov State Medical University, Kharkov; G Kyselyova, Crimean Republican AIDS centre, Simferopol; M Sluzhynska, Lviv Regional HIV/AIDS Prevention and Control CTR, Lviv.

United Kingdom: (B Gazzard), St. Stephen's Clinic, Chelsea and Westminster Hospital, London; AM Johnson, E Simons, S Edwards, Mortimer Market Centre, London; A Phillips, MA Johnson, A Mocroft, Royal Free and University College Medical School, London (Royal Free Campus); C Orkin, Royal London Hospital, London; J Weber, G Scullard, Imperial College School of Medicine at St. Mary's, London; A Clarke, Royal Sussex County Hospital, Brighton; C Leen, Western General Hospital, Edinburgh.

The following centers have previously contributed data to EuroSIDA:

Infectious Diseases Hospital, Sofia, Bulgaria.

Hôpital de la Croix Rousse, Lyon, France.

Hôpital de la Pitié‐Salpétière, Paris, France.

Unité INSERM, Bordeaux, France.

Hôpital Edouard Herriot, Lyon, France.

Bernhard Nocht Institut für Tropenmedizin, Hamburg, Germany.

1st I.K.A Hospital of Athens, Athens, Greece.

Ospedale Riuniti, Divisione Malattie Infettive, Bergamo, Italy.

Ospedale di Bolzano, Divisione Malattie Infettive, Bolzano, Italy.

Ospedale Cotugno, III Divisione Malattie Infettive, Napoli, Italy.

Dérer Hospital, Bratislava, Slovakia.

Hospital Carlos III, Departamento de Enfermedades Infecciosas, Madrid, Spain.

Kiev Centre for AIDS, Kiev, Ukraine.

Luhansk State Medical University, Luhansk, Ukraine.

Odessa Region AIDS Center, Odessa, Ukraine.

### HivBivus (Sweden)

Central coordination: L Morfeldt, G Thulin, A Sundström.

Participating physicians (city): B Åkerlund (Huddinge); K Koppel, A Karlsson (Stockholm); L Flamholc, C Håkangård (Malmö).

### The ICONA Foundation (Italy)

#### Board of Directors

A d'Arminio Monforte (President), A Antinori, A Castagna, F Castelli, R Cauda, G Di Perri, M Galli, R Iardino, G Ippolito, GC Marchetti, CF Perno, F von Schloesser, P Viale

#### Scientific secretary

A d'Arminio Monforte, A Antinori, A Castagna, F Ceccherini‐Silberstein, A Cozzi‐Lepri, E Girardi, S Lo Caputo, C Mussini, M Puoti

#### Steering Committee

M Andreoni, A Ammassari, A Antinori, C Balotta, A Bandera, P Bonfanti, S Bonora, M Borderi, A Calcagno, L Calza, MR Capobianchi, A Castagna, F Ceccherini‐Silberstein, A Cingolani, P Cinque, A Cozzi‐Lepri, A d'Arminio Monforte, A De Luca, A Di Biagio, E Girardi, N Gianotti, A Gori, G Guaraldi, G Lapadula, M Lichtner, S Lo Caputo, G Madeddu, F Maggiolo, G Marchetti, S Marcotullio, L Monno, C Mussini, S Nozza, M Puoti, E Quiros Roldan, R Rossotti, S Rusconi, MM Santoro, A Saracino, M Zaccarelli.

#### Statistical and Monitoring Team

A Cozzi‐Lepri, I Fanti, L Galli, P Lorenzini, A Rodano, M Shanyinde, A Tavelli

#### Biological Bank INMI

F Carletti, S Carrara, A Di Caro, S Graziano, F Petrone, G Prota, S Quartu, S Truffa

#### Participating Physicians and Centers

Italy A Giacometti, A Costantini, V Barocci (Ancona); G Angarano, L Monno, C Santoro (Bari); F Maggiolo, C Suardi (Bergamo); P Viale, V Donati, G Verucchi (Bologna); F Castelli, C Minardi, E Quiros Roldan (Brescia); T Quirino, C Abeli (Busto Arsizio); PE Manconi, P Piano (Cagliari); B Cacopardo, B Celesia (Catania); J Vecchiet, K Falasca (Chieti); A Pan, S Lorenzotti (Cremona); L Sighinolfi, D Segala (Ferrara); F Mazzotta, F Vichi (Firenze); G Cassola, C Viscoli, A Alessandrini, N Bobbio, G Mazzarello (Genova); C Mastroianni, V Belvisi (Latina); P Bonfanti, I Caramma (Lecco); A Chiodera, P Milini (Macerata); A d'Arminio Monforte, M Galli, A Lazzarin, G Rizzardini, M Puoti, A Castagna, G Marchetti, MC Moioli, R Piolini, AL Ridolfo, S Salpietro, C Tincati, (Milano); C Mussini, C Puzzolante (Modena); A Gori, G Lapadula (Monza); N Abrescia, A Chirianni, G Borgia, R Orlando, G Bonadies, F Di Martino, I Gentile, L Maddaloni (Napoli); AM Cattelan, S Marinello (Padova); A Cascio, C Colomba (Palermo); F Baldelli, E Schiaroli (Perugia); G Parruti, F Sozio (Pescara); G Magnani, MA Ursitti (Reggio Emilia); M Andreoni, A Antinori, R Cauda, A Cristaudo, V Vullo, R Acinapura, G Baldin, M Capozzi, S Cicalini, A Cingolani, L Fontanelli Sulekova, G Iaiani, A Latini, I Mastrorosa, MM Plazzi, S Savinelli, A Vergori (Roma); M Cecchetto, F Viviani (Rovigo); G Madeddu, P Bagella (Sassari); A De Luca, B Rossetti (Siena); A Franco, R Fontana Del Vecchio (Siracusa); D Francisci, C Di Giuli (Terni); P Caramello, G Di Perri, S Bonora, GC Orofino, M Sciandra (Torino); M Bassetti, A Londero (Udine); G Pellizzer, V Manfrin (Vicenza), G Starnini, A Ialungo (Viterbo).

### Nice HIV Cohort (France)

Central coordination: C Pradier*, E Fontas, K Dollet, C Caissotti.

Participating physicians: P Dellamonica, E Bernard, J Courjon, E Cua, F De Salvador‐Guillouet, J Durant, C Etienne, S Ferrando, V Mondain‐Miton, A Naqvi, I Perbost, S Pillet, B Prouvost‐Keller, P Pugliese, V Rio, K Risso, PM Roger.

### SHCS (Swiss HIV Cohort Study, Switzerland)

The data are gathered by the Five Swiss University Hospitals, two Cantonal Hospitals, 15 affiliated hospitals and 36 private physicians (listed in http://www.shcs.ch/180-health-care-providers).

#### Members of the Swiss HIV Cohort Study

V Aubert, M Battegay, E Bernasconi, J Böni, DL Braun, Hc Bucher , A Calmy, M Cavassini, A Ciuffi, G Dollenmaier, M Egger, L Elzi, J Fehr, J Fellay, H Furrer (Chairman of the Clinical and Laboratory Committee), CA Fux, HF Günthard (President of the SHCS), D Haerry (deputy of “Positive Council”), B Hasse, HH Hirsch, M Hoffmann, I Hösli, C Kahlert, L Kaiser, O Keiser, T Klimkait, RD Kouyos, H Kovari,B Ledergerber, G Martinetti, B Martinez de Tejada, C Marzolini, KJ Metzner, N Müller, D Nicca, G Pantaleo, P Paioni, A Rauch (Chairman of the Scientific Board), C Rudin (Chairman of the Mother & Child Substudy), AU Scherrer (Head of Data Centre), P Schmid, R Speck, M Stöckle, P Tarr, A Trkola, P Vernazza, G Wandeler, R Weber*, S Yerly.

## Financial acknowledgements

The D:A:D study was supported by a grant [grant number DNRF126] from the Danish National Research Foundation (CHIP & PERSIMUNE); the Highly Active Antiretroviral Therapy Oversight Committee (HAARTOC), a collaborative committee with representation from academic institutions, the European Agency for the Evaluation of Medicinal Products, the United States Food and Drug Administration, the patient community, and pharmaceutical companies with licensed anti‐HIV drugs in the European Union: AbbVie, Bristol‐Myers Squibb, Gilead Sciences Inc., ViiV Healthcare, Merck & Co Inc. and Janssen Pharmaceuticals. Supported also by a grant from the Dutch Ministry of Health, Welfare and Sport through the Center for Infectious Disease Control of the National Institute for Public Health and the Environment to Stiching HIV Monitoring (ATHENA); by a grant from the Agence nationale de recherches sur le sida et les hépatites virales [ANRS, Action Coordonnée no. 7, Cohortes] to the Aquitaine Cohort; The Australian HIV Observational Database (AHOD) is funded as part of the Asia Pacific HIV Observational Database, a program of The Foundation for AIDS Research, amfAR, and is supported in part by a grant from the U.S. National Institutes of Health's National Institute of Allergy and Infectious Diseases (NIAID) [grant number U01‐AI069907] and by unconditional grants from Merck Sharp & Dohme; Gilead Sciences; Bristol‐Myers Squibb; Boehringer Ingelheim; Janssen‐Cilag; ViiV Healthcare. The Kirby Institute is funded by The Australian Government Department of Health and Ageing, and is affiliated with the Faculty of Medicine, The University of New South Wales; by grants from the Fondo de Investigación Sanitaria [grant number FIS 99/0887] and Fundación para la Investigación y la Prevención del SIDA en Espanã [grant number FIPSE 3171/00], to the Barcelona Antiretroviral Surveillance Study (BASS); by the National Institute of Allergy and Infectious Diseases, National Institutes of Health [grants number 5U01AI042170‐10, 5U01AI046362‐03], to the Terry Beirn Community Programs for Clinical Research on AIDS (CPCRA); by primary funding provided by the European Union's Seventh Framework Programme for research, technological development and demonstration under EuroCoord grant agreement n˚ 260694 and unrestricted grants by Bristol‐Myers Squibb, Janssen R&D, Merck and Co. Inc., Pfizer Inc., GlaxoSmithKline LLC, (the participation of centres from Switzerland is supported by The Swiss National Science Foundation (Grant 108787)) to the EuroSIDA study; by unrestricted educational grants of AbbVie, Bristol‐Myers Squibb, Gilead Sciences, GlaxoSmithKline, Pfizer, Janssen Pharmaceuticals to the Italian Cohort Naive to Antiretrovirals (The ICONA Foundation); and financed within the framework of the Swiss HIV Cohort Study, supported by the Swiss National Science Foundation (grant #148522) and by the SHCS research foundation.
